# Case Report: Reconstruction of Medialis Malleolus (1/4 of the Ankle Joint) After Resection of Distal Tibia Tumor With an Uncemented Three-Dimensional-Printed Prosthesis

**DOI:** 10.3389/fsurg.2022.844334

**Published:** 2022-03-24

**Authors:** Shang Wang, Yi Luo, Yuqi Zhang, Yitian Wang, Chuanxi Zheng, Chongqi Tu, Yong Zhou

**Affiliations:** Department of Orthopedics, West China Hospital, Sichuan University, Chengdu, China

**Keywords:** medialis malleolus, joint preservation, 3D-printed endoprosthesis, case report, fibrosarcoma

## Abstract

**Introduction:**

Few patients presented with a distal tibial tumor that only invaded a small area of bone in the medial malleolus. There have been no previous cases in which only the medial or lateral malleolus was removed and reconstruction was complete. This article describes our attempt to reconstruct the medial malleolus (1/4 of the ankle joint) after resection of a distal tibial tumor with an uncemented three-dimensional (3D)-printed prosthesis.

**Case Description:**

A 39-year-old man presented with a lump in the right medial malleolus, and biopsy results suggested fibrosarcoma. To preserve the patient's normal bone and function, we only removed the medial malleolus and reconstructed the ankle joint using a personalized 3D-printed prosthesis. The patient had no complications other than necrosis of the skin flap that covered the wound. The patient recovered well after undergoing an additional skin flap transfer. Follow-up at 7 months and again at 3 years after surgery showed good ankle function and stability, with no pain or complications.

**Conclusion:**

The 3D-printed partial ankle prosthesis had a good matching degree, strength, and osseointegration ability, but also had a few complications. The patient achieved satisfactory ankle function and stability. However, a longer follow-up period is needed, and more research is required to confirm the efficacy of the prosthesis.

## Introduction

Bone and soft tissue tumors in the distal tibia are rare and easy to diagnose early because of the lack of soft tissue coverage (1–3). The prognosis for these patients is good, and they are expected to survive for a long time after treatment. To improve the quality of life for these patients after surgery, it is important to preserve joint function. In recent decades, ablation has been the traditional treatment for these patients (4). Limb-sparing treatment is currently the main treatment for most cases owing to advanced diagnostic and surgical techniques (3, 5).

At present, the surgical methods commonly used include arthrodesis of the ankle, osteoarticular reconstruction using an autograft or allograft, and prosthetic replacement or bone transport (1, 4). However, there is no consensus on the best treatment for tumors of the distal tibia as every method has its advantages and disadvantages. For example, ankle arthrodesis achieves good joint stability while compromising joint function, and operations that preserve the ankle joint to retain good joint function might result in more complications.

The treatment method should be determined using a patient-centered approach. For patients with a large tumor, we need to remove the epiphysis and articular surface if the tumor has affected them. However, in some patients like ours, the tumor invaded only a small area of the bony structure. We hoped to only remove the involved bone with the standard resection boundary to preserve as much normal bone as possible. It is almost impossible for traditional surgical methods to achieve this because it is difficult to accurately reconstruct the ankle joint at the osteotomy site due to the shape of the defect (6). According to the results of the literature search, there have been no previous cases in which only the medial or lateral malleolus was removed, and reconstruction was complete. Herein, we describe our attempt to reconstruct the medial malleolus (1/4 of the ankle joint) after resection of a distal tibial tumor with an uncemented three-dimensional (3D)-printed prosthesis.

## Case Description

### History

**Figure 1:**
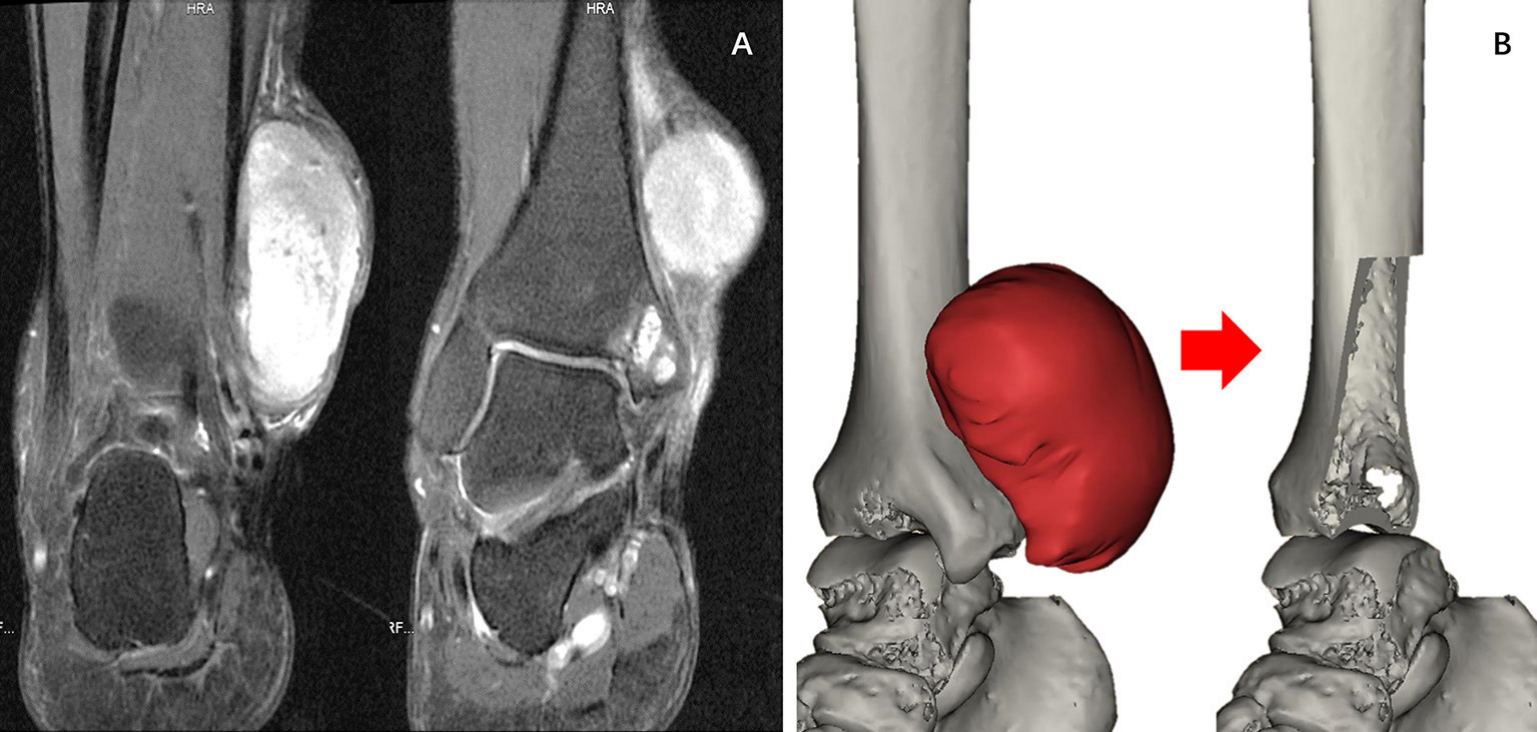
**(A)** Magnetic resonance imaging. Soft tissue tumors can be seen on the anterior medial side of the ankle joint, and the ankle joint is slightly involved. **(B)** Reconstructed 3D models of the extent of the resection and the size of the ankle joint defect.

A 39-year-old man presented with a lump in the right medial malleolus of 11 months duration. The diameter of the lump gradually increased from ~1–6 cm, and the lump caused obvious pain (Figure 1A). He visited our institution in January 2019 and underwent a biopsy. The biopsy results suggested fibrosarcoma [French Federation of Cancer Centers (FNCLCC) grade 3, and Enneking grade IIB]. Physical examination revealed a tough mass with a clear demarcation from the surrounding soft tissues and significant pain on the application of pressure. The mobility of the right ankle and muscle strength and sensation in the right foot were not affected. In March 2019, after two cycles of chemotherapy, the lump had softened, but had not changed in size. As the tumor invaded only a small amount of bone within the medial malleolus and did not metastasize, we planned to only remove the medial malleolus with the standard resection boundary and customize a personalized 3D-printed prosthesis for replacement, to preserve the patient's normal bone and function.

### Prosthesis Design and Manufacture

**Figure 2:**
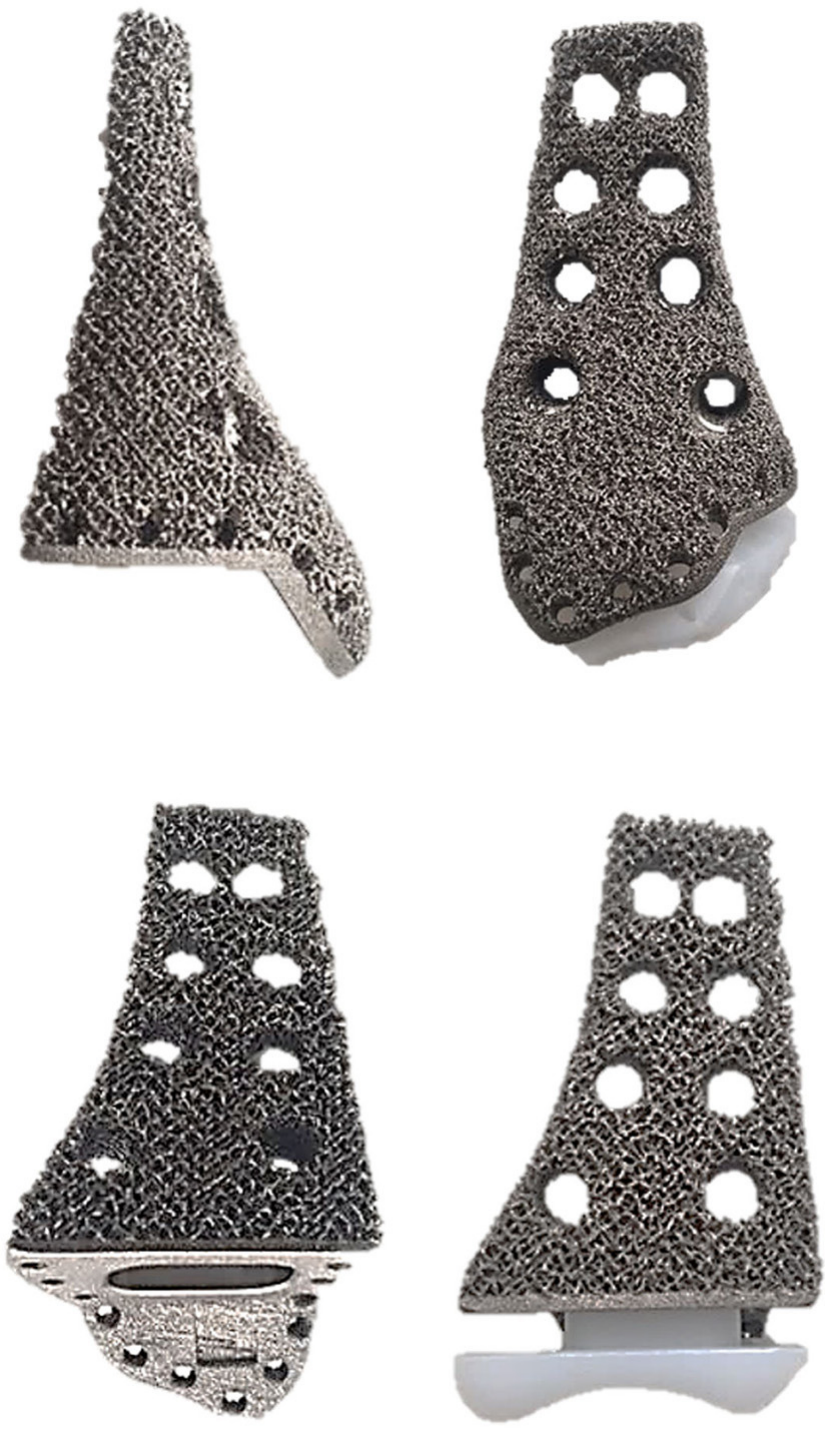
Final product of 3D-printed prosthesis.

Our clinical team designed the prosthesis, which was manufactured by Chunli Co., Ltd., Tongzhou, Beijing, China. The goal of our design process was to improve the matching degree of the prosthesis and maximize the recovery of joint function. In the first step, we built virtual 3D tibia models based on computerized tomography (CT) data using Materialize Mimics V20.0 (Materialize Corp., Leuven, Belgium) software. We then built a virtual tumor model using an image fusion technique integrating magnetic resonance imaging data. We determined the osteotomy plane based on the model (Figure 1B). In the second step, we designed a preliminary model of the prosthesis. The shape of the endoprosthesis was optimized *via* computer simulation to ensure a satisfactory fit with the tibia. The surgical approach and the specific anatomy of the patient's osteotomy determined the position of the eight screw holes and suture holes that were added to enable us to obtain a stable fixation. The surface of the prosthesis was designed as a trabecular structure with porosity to facilitate the formation of a well-integrated implant–bone interface. The articular surface was designed to maximize joint function. To reduce the wear of the prosthesis and protect the talus, a portion of the articular surface was made of polyethylene. The endoprosthesis was made of a titanium alloy (Ti6Al4V) and fabricated using the electron beam melting technique using an Arcam EBM Q10plus machine (Arcam EBM, Mölndal, Sweden) (Figure 2). The yield strength of the prosthesis is about 550 Mpa, according to mechanical testing.

### Surgery

**Figure 3:**
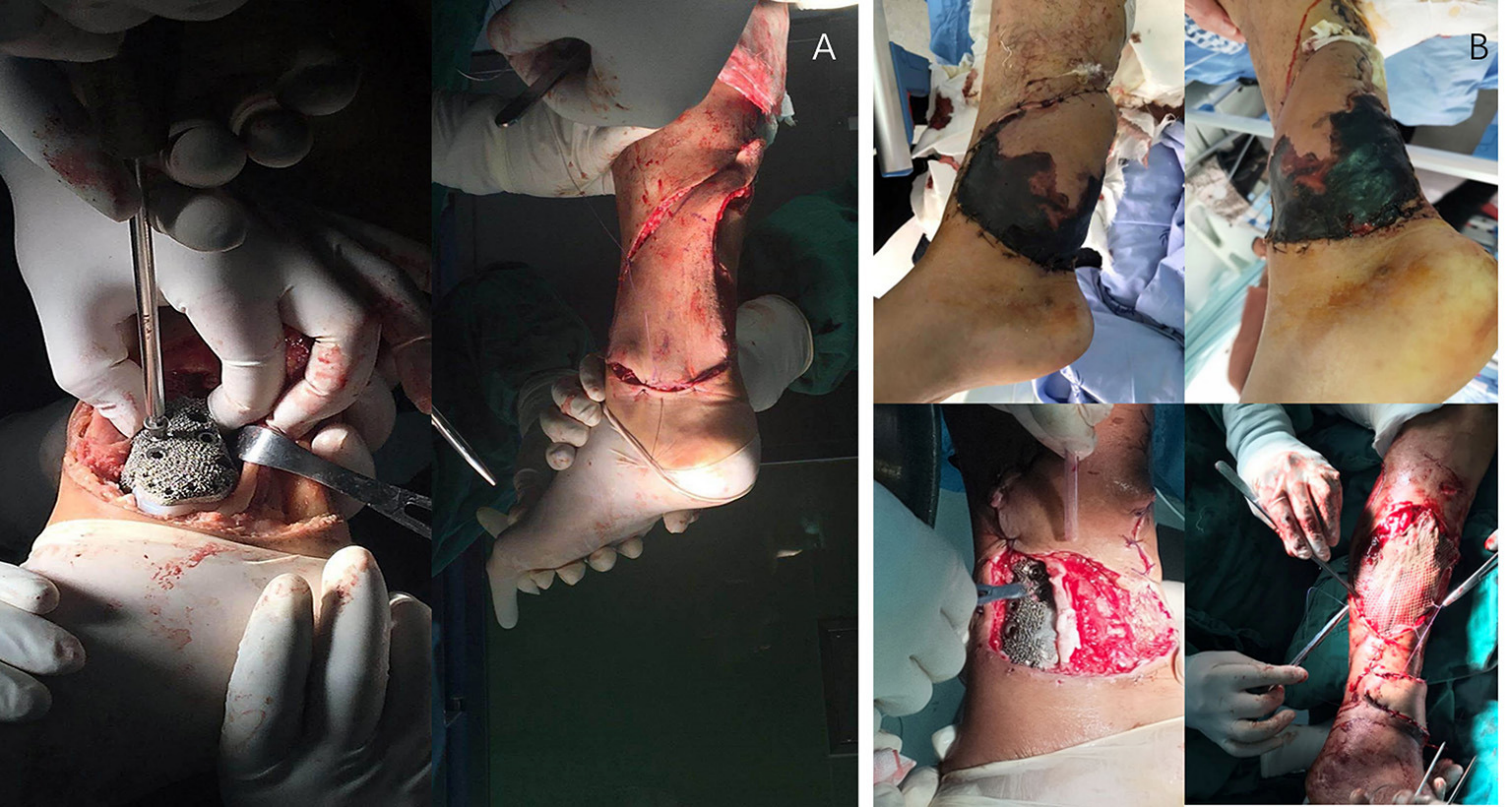
**(A)** Prosthesis implantation and flap transfer. **(B)** Postoperative skin necrosis and the second skin flap transplantation.

The senior surgeon at our institution (C. T.) performed the surgery. According to the preoperative plan, the osteotomy length was measured with Vernier calipers and marked. The proximal osteotomy was performed first, followed by the lateral osteotomy. The custom-designed 3D-printed prosthesis was implanted into the osteotomy site and fixed with six screws. Owing to the presence of small gaps, the uninvaded bone cortex of the resected portion of the tibia was filled with autogenous bone between the upper end of the prosthesis and the host bone to assist bone healing. The deltoid ligament was repaired by fixing it to the surface of the prosthesis. As the tumor resection caused a large area of skin and soft tissue defects on the surface of the prosthesis, we performed transposition of the peroneal nerve *via* a pedicled fascia flap to cover the surface of the prosthesis and, used a vacuum-assisted closure (Figure 3A). The patient underwent necrotic tissue debridement 15 days after the first surgery, repeat transposition of the skin flap, and skin grafting 21 days after the first surgery due to partial epidermal necrosis on the surface of the skin flap. The last two surgeries were performed by microsurgical experts at our institution (Figure 3B).

### Outcome

**Figure 4:**
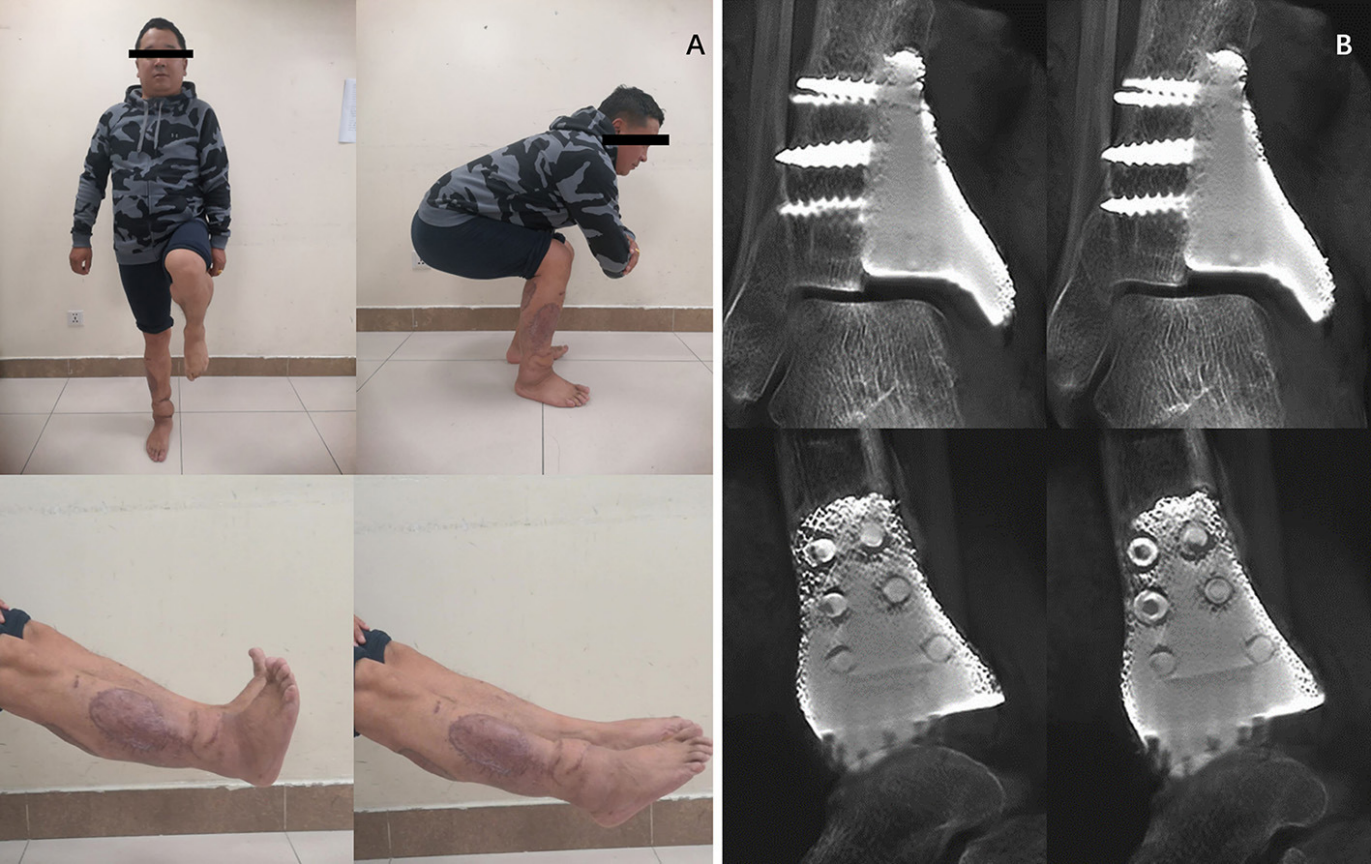
**(A)** Digital tomosynthesis showed that the 3D prosthesis tightly integrated with the ankle joint, and partial bone substance could be observed to grow into the porous of the prosthesis. **(B)** Recovery of skin flap and functional outcomes of the ankle joint 7 months after surgery.

The patient was hospitalized for 37 days and underwent three surgeries. After the last operation, the patient's skin flap and surrounding skin recovered well, and the patient was discharged from the hospital. The patient's right ankle function was satisfactory seven months postoperatively. Dorsiflexion and plantar flexion of the right ankle joint were 20° and 45°, respectively. The range of motion for inversion and eversion was 30°. The ranges of motion of the left and right ankles were comparable, with the exception of eversion, which was slightly less than that of the left ankle. The patient could walk independently and had a normal gait (Figure 4A). The patient did not complain of any pain in the ankle or surrounding area. Radiography showed that the prosthesis fits well with the distal tibia, and the position of the prosthesis was normal. No obvious complications were observed. The tomosynthesis-Shimadzu metal artifact reduction technology scan showed that the 3D-printed prosthesis was well integrated with the distal tibia (Figure 4B), The Musculoskeletal Tumor Society (MSTS) score was 96.6% (29/30). Three years after the surgery, the patient was not able to return to our institution for review. Therefore, we followed up with the patient telephonically. Examination at the local hospital did not reveal any obvious signs of tumor recurrence, and the patient reported no complications during the 3-year period. The patient's ankle function was the same as that at 7 months postoperatively, and the patient was able to perform daily activities and work independently.

## Discussion

Tumors of the distal tibia have a low incidence and are easily found in the early stages. Mavrogenis et al. (5) found that patients who underwent limb salvage or amputation had similar rates of survival, local recurrence, and complications, but patients who underwent limb salvage surgery had a better function.

Currently, the main methods of limb salvage therapy include arthrodesis of the ankle, osteoarticular reconstruction using an allograft or autograft, and non-biological reconstruction (arthroplasty) (7, 8). The main complications include graft fracture, proximal osteotomy non-union, and infection.

Arthrodesis can obtain adequate stability, and patients can bear weight early, but there are limitations in functional ability and gait (1, 9). Following the resection of the distal tibia, arthrodesis of the ankle often needs the use of suitable bone grafts and fixation. After arthrodesis, patients completely lose the ability to move their ankles, and this produces corresponding complications. Campanacci et al. (1) reported the mean MSTS scores of eight arthrodesis patients as 60%. The use of bone grafts increases the risk of prosthetic infection and non-union (4, 10).

Patients who undergo joint reconstruction generally have better function. Niimi et al. (11) reported on six patients receiving autografts, and their mean MSTS scores were 84.5%. In the report by Xu et al. (7), all six patients presented with plantar hyperflexion, and the MSTS scores were 74.3 ± 4.4%. Osteoarticular reconstruction using an autograft has many advantages, including the ability to reattach soft tissues to the bone, a prosthesis that is easy to obtain, and the absence of immunologic response. In the article by Shekkeris et al. (12), the mean functional outcome score (MSTS) for the four patients with a surviving endoprosthesis was 70%. Natarajan et al. (13) performed prosthetic replacement of the distal tibia and ankle in six patients, and their mean MSTS score was 80%. Joint reconstructions have more complications than arthrodesis, such as flap complications, loosening of the prosthesis, and fracture of the prosthesis. Moreover, the structural integrity of the involved bone must be maintained (11, 13–15).

For patients whose resection boundary does not reach the whole joint, the traditional surgical methods cannot retain the healthy side of the ankle joint. This was the case for our patient. Therefore, we designed a surgical scheme in which only the medial malleolus was replaced with a 3D-printed prosthesis. Our approach retained the tibia and ankle joint of the healthy side and simultaneously reconstructed the tibia and ankle joint after resection. In the present case, the patient's symptoms improved significantly. Prosthesis-related complications were not observed. After postoperative recovery, the patient's ankle function was close to normal, and he was able to walk, run, and complete other functions. Imaging showed that the osteotomy had healed well, and the prosthesis was stable and in the correct position.

A 3D-printed prosthesis can perfectly match the patient's bone defect after resection and can retain the bone mass as much as possible because it is specially designed according to the anatomy of the patient's osteotomy. In addition, 3D-printed prostheses have sufficient mechanical strength, and their sophisticated porous structure facilitates the formation of a well-integrated implant–bone interface. Some institutions have applied this technique in orthopedic surgery for areas, including the pelvis, scapula, clavicle, and proximal tibia. The joint function of these patients was satisfactory and there were no prosthesis-related complications (16–19).

The study had some limitations. A longer time to follow up is needed to obtain more accurate treatment results and more convincing data, and one case is not sufficient to illustrate the efficacy of 3D-printed partial ankle prostheses. However, our case provides a basis for the treatment of this type of disease.

## Conclusions

In our case, the 3D-printed partial ankle prosthesis had a good therapeutic outcome in patients who retained a healthy tibia and ankle joint. We believe that this type of prosthesis is not only suitable for patients with tumors, but also for bone defects caused by inflammation and trauma. At present, the prosthesis appears to have a good matching degree, strength, and osseointegration ability, but has few complications. The patient achieved satisfactory ankle function after reconstruction with the 3D-printed partial prosthesis. However, a longer follow-up period is needed, and more research is required to confirm the efficacy of the prosthesis.

## Data Availability Statement

The original contributions presented in the study are included in the article/supplementary material, further inquiries can be directed to the corresponding authors.

## Ethics Statement

Written informed consent was obtained from the individual(s) for the publication of any potentially identifiable images or data included in this article.

## Author Contributions

SW, YL, YZho, and CT were involved with the concept and design of this manuscript. SW, YZho, YW, and CZ were involved with the acquisition of subjects and data. YL, YZha, and CT were involved in the design of the endoprosthesis and writing and revision of the manuscript. All Authors contributed to the article and approved the submitted version.

## Funding

This work was supported, in part, by the Technology Research Program of Sichuan Province (2020YFS0036), 1.3.5 project for disciplines of excellence–Clinical Research Incubation Project, West China Hospital, Sichuan University (2020HXFH004), Sichuan Science and Technology Innovation Team of China (2019JDTD0008), 1.3.5 project for disciplines of excellence, West China Hospital, Sichuan University (ZYJC18036).

## Conflict of Interest

The authors declare that the research was conducted in the absence of any commercial or financial relationships that could be construed as a potential conflict of interest.

## Publisher's Note

All claims expressed in this article are solely those of the authors and do not necessarily represent those of their affiliated organizations, or those of the publisher, the editors and the reviewers. Any product that may be evaluated in this article, or claim that may be made by its manufacturer, is not guaranteed or endorsed by the publisher.
